# Ophthalmologic manifestations in Taiwanese patients with mucopolysaccharidoses

**DOI:** 10.1002/mgg3.617

**Published:** 2019-03-08

**Authors:** Hsiang‐Yu Lin, Wei‐Chun Chan, Lee‐Jen Chen, Yuan‐Chieh Lee, Shu‐I Yeh, Dau‐Ming Niu, Pao Chin Chiu, Wen‐Hui Tsai, Wuh‐Liang Hwu, Chih‐Kuang Chuang, Shuan‐Pei Lin

**Affiliations:** ^1^ Department of Medicine Mackay Medical College New Taipei City Taiwan; ^2^ Department of Pediatrics Mackay Memorial Hospital Taipei Taiwan; ^3^ Department of Medical Research Mackay Memorial Hospital Taipei Taiwan; ^4^ Mackay Junior College of Medicine, Nursing and Management Taipei Taiwan; ^5^ Department of Medical Research China Medical University Hospital China Medical University Taichung Taiwan; ^6^ Institute of Biomedical Sciences Mackay Medical College New Taipei City Taiwan; ^7^ Department of Ophthalmology Mackay Memorial Hospital Taipei Taiwan; ^8^ Department of Ophthalmology Buddhist Tzu Chi General Hospital Hualien Taiwan; ^9^ Department of Pediatrics Taipei Veterans General Hospital Taipei Taiwan; ^10^ Department of Pediatrics Kaohsiung Veterans General Hospital Kaohsiung Taiwan; ^11^ Department of Pediatrics Chi Mei Medical Center Tainan Taiwan; ^12^ Department of Pediatrics National Taiwan University Hospital Taipei Taiwan; ^13^ College of Medicine Fu‐Jen Catholic University Taipei Taiwan; ^14^ Department of Infant and Child Care National Taipei University of Nursing and Health Sciences Taipei Taiwan

**Keywords:** corneal opacity, hyperopia, mucopolysaccharidosis, ocular hypertension, visual acuity

## Abstract

**Background:**

Mucopolysaccharidoses (MPSs) are a group of rare lysosomal storage disorders characterized by the accumulation of glycosaminoglycans in various tissues and organs. Ocular problems that affect the cornea, trabecular meshwork, sclera, retina, and optic nerve are very common in these patients. However, there was limited literature focusing on comprehensive ocular findings in different types of MPS.

**Methods:**

We retrospectively reviewed the clinical ophthalmologic features and electrodiagnostic results of 50 Taiwanese patients with a diagnosis of MPS (34 males and 16 females; age range, 1.1–34.9 years; nine with MPS I, 17 with MPS II, 17 with MPS IV, and seven with MPS VI).

**Results:**

Among 44 patients with available data for visual acuity, 15 patients (34%) had a visual acuity of less than 0.5 (6/12) equivalent in their better eye, including 71% of those with MPS VI, 38% with MPS IV, 29% with MPS I, and 14% with MPS II. Severe corneal opacities existed in 57% of MPS VI patients and 11% of MPS I patients, compared with none for MPS II and MPS IV patients. Among 80 eyes with available data of refraction, 11 eyes (14%) had myopia (≦−0.50 D), 55 eyes (69%) had hyperopia (≧0.50 D), and 55 eyes (69%) had high astigmatism (≧1.50 D). Ocular hypertension was found in 45% (28/62) of eyes. There were 16% (14/90), 11% (10/90), 13% (12/90), 31% (27/86), and 79% (30/38) of MPS eyes with lens opacities, optic disc swelling, optic disc cupped, retinopathy, and visual pathway dysfunction, respectively. Intraocular pressure was positively correlated with the severity of corneal opacity (*p *<* *0.01).

**Conclusions:**

Ocular complications with significant reduction in visual acuity are common in MPS patients. Diagnostic problems may arise in these patients with severe corneal opacification, especially for those with MPS VI and MPS I.

## 1 INTRODUCTION

Mucopolysaccharidoses (MPSs; OMIM252700) consist of a group of rare genetic disorders caused by deficiencies in specific lysosomal enzymes involved in the sequential degradation of glycosaminoglycans (GAGs) which accumulate in various cells and tissues, leading to progressive multi‐organ dysfunction. Seven distinct types of MPS disorders (I, II, III, IV, VI, VII, and IX) with 11 specific lysosomal enzyme deficiencies have been reported. The clinical manifestations of MPS are progressive and chronic with a wide spectrum of clinical severity and prognosis among the different types (Chuang & Lin, 2007; Neufield & Muenzer, 2001). The clinical presentation in patients with MPS includes vision and hearing impairment, coarse facial features, airway obstruction, cardiopulmonary impairment, organomegaly, developmental delay, short stature, joint rigidity, and skeletal deformities (dysostosis multiplex). All types of MPS have an autosomal recessive mode of inheritance except for MPS II (Hunter syndrome), which is transmitted as an X‐linked recessive manner and thus primarily affects males. The incidence of MPS is estimated to be 1.9‐4.5/100,000 live births (Lin et al., 2009).

Ocular problems are very common in patients with MPS with the involvement of the cornea, trabecular meshwork, lens, optic disc, retina, sclera, and optic nerve (Ashworth, Biswas, Wraith, & Lloyd, 2006a). Corneal clouding results from GAG deposition in all layers of the cornea, including epithelium, keratocytes, stroma, and endothelium, both intracellularly and extracellularly, leading to disrupted arrangement of collagen fibrils (Summers & Ashworth, 2011). Ocular hypertension and glaucoma occur secondary to GAG‐mediated obstruction outflow through the trabecular meshwork (open‐angle glaucoma) or narrowing of the anterior chamber angle (angle‐closure glaucoma) (Ferrari et al., 2011). Optic disc swelling (i.e., papilloedema) and subsequent optic nerve atrophy can occur as a result of high intracranial pressure, or nerve compression by GAG‐thickened sclera and dura, or intracellular GAG deposition within optic nerve ganglion cells (Ashworth et al., 2010). Retinopathy occurs because of GAG deposition within retinal pigment epithelial cells and in the photoreceptor matrix, leading to progressive photoreceptor loss, retinal degeneration, and dysfunction (Ganesh, Bruwer, & Al‐Thihli, 2013).

Here, we determined the prevalence and severity of ocular complications in a group of 50 Taiwanese MPS patients before ophthalmologic surgery, enzyme replacement therapy or hematopoietic stem cell transplantation (HSCT). We also evaluated the relationship between each ophthalmologic manifestation and different types of MPS.

## 2 MATERIALS AND METHODS

### 2.1 Ethical compliance

The study protocol was approved by the Ethics Committee of Mackay Memorial Hospital, and written informed consent was provided by a parent of the children and from the patients themselves if they were over 18 years of age.

### 2.2 Study population

We retrospectively reviewed the medical records, clinical ophthalmologic features and electrodiagnostic results of 50 Taiwanese patients with a diagnosis of MPS (34 males and 16 females; mean age: 14.3 ± 8.5 years; median age, 13.4 years; age range, 1.1–34.9 years; nine with MPS I, 17 with MPS II, 17 with MPS IV, and seven with MPS VI) at Mackay Memorial Hospital between January 1996 and December 2017. The diagnosis of the type of MPS was confirmed by specific enzyme activity assays in serum, leukocytes and/or skin fibroblasts, two‐dimensional electrophoresis of urinary GAGs, and/or identification of a pathogenic mutation (Chuang, Lin, & Chung, 2001). None had received ophthalmologic surgery, enzyme replacement therapy or HSCT at the time of the study.

### 2.3 Ophthalmologic assessments

Ophthalmologic examinations, including visual acuity, refractive errors (e.g., myopia, hyperopia, and astigmatism), lens opacity, and intraocular pressure (IOP) measurement (if possible) were documented. The examinations comprised assessment of best‐corrected visual acuity by Snellen charts, measurement of IOP by noncontact air‐puff tonometry (TX‐F, Canon) in the clinic, slit‐lamp examination of the anterior segment (Haag Streit BQ 900; Köniz, Switzerland), as well as direct and indirect funduscopy. The degree of corneal opacity was subjectively graded by one observer (WCC) as mild (+), moderate (++), or severe (+++). For refractive error examinations by the use of an autorefractor, myopia was defined as sphere power ≦−0.50 D, hyperopia was defined as sphere power ≧0.50 D, and high astigmatism was defined as cylinder power ≧1.50 D (Lai, Hsu, Wang, Chang, & Chang, 2010). The results of IOP were classified as normal (≦21 mmHg) or ocular hypertension (>21 mmHg). Severe ocular hypertension was defined as IOP > 30 mmHg (Ashworth, Biswas, Wraith, & Lloyd, 2006b). The appearance of the optic disc was recorded as normal, atrophic, swelling, or cupped if possible visualization. The presence of retinopathy was detected by dilated fundal examination of the retina. Visual evoked potentials (VEPs) were performed in patients of suspected visual pathway dysfunction. The VEPs were recorded using a Reporter Analysis System (Reporter, EsaOteBiomedica, Florence, Italy). The testing protocol of VEPs at our institution was performed according to that of the previous report by (Suppiej et al. (2013). We used the flash VEPs in the present study. The peak latency of the P2 wave and the amplitude were analyzed in Oz location.

### 2.4 Data analysis and statistics

All results were calculated using descriptive statistics, including numbers and percentages for categorical variables, as well as mean, median, and range (minimum and maximum values) for continuous variables. We compared ocular characteristics among different types of MPS. The relationship between IOP and severity of corneal opacity was determined using Pearson's correlation coefficient (*r*), and significance was tested using Fisher's *r–z* transformations. Two‐tailed *p*‐values were computed. All statistical analyses were performed using SPSS version 11.5 (SPSS Inc, Chicago, Illinois), and differences with *p *<* *0.05 were considered statistically significant.

## 3 RESULTS

Table 1 shows the ocular problems of Taiwanese patients with different types of MPS in this study. Tables 2, 3, 4, 5 show the demographic data and ophthalmologic characteristics of 50 Taiwanese patients with MPS I, II, IV, and VI, respectively. The age ranges of patients with MPS I, II, IV, and VI were 1.6–34.9, 3.3–34.3, 1.1–29.4, and 8.3–25.6 years, respectively.

**Table 1:** Ocular problems of Taiwanese patients with mucopolysaccharidosis. D, diopters

	MPS I	MPS II	MPS IV	MPS VI	All
Visual acuity (better eye < 0.5) (44 patients)	29% (*n* = 7)	14% (*n* = 14)	38% (*n* = 16)	71% (*n* = 7)	34% (*n* = 44)
Amblyopia (100 eyes)	28% (*n* = 18)	6% (*n* = 34)	15% (*n* = 34)	71% (*n* = 14)	22% (*n* = 100)
Corneal clouding (100 eyes)	100% (*n* = 18)	0% (*n* = 34)	94% (*n* = 34)	100% (*n* = 14)	64% (*n* = 100)
Myopia (≦−0.50 D) (80 eyes)	0% (*n* = 12)	11% (*n* = 28)	25% (*n* = 32)	0% (*n* = 8)	14% (*n* = 80)
Hyperopia (≧0.50 D) (80 eyes)	92% (*n* = 12)	68% (*n* = 28)	53% (*n* = 32)	100% (*n* = 8)	69% (*n* = 80)
High astigmatism (≧1.50 D) (80 eyes)	83% (*n* = 12)	61% (*n* = 28)	72% (*n* = 32)	63% (*n* = 8)	69% (*n* = 80)
Ocular hypertension (62 eyes)	33% (*n* = 12)	55% (*n* = 20)	31% (*n* = 16)	64% (*n* = 14)	45% (*n* = 62)
Lens opacity (90 eyes)	38% (*n* = 16)	18% (*n* = 34)	6% (*n* = 34)	0% (*n* = 6)	16% (*n* = 90)
Optic disc swelling (90 eyes)	17% (*n* = 18)	13% (*n* = 32)	0% (*n* = 34)	50% (*n* = 6)	11% (*n* = 90)
Optic disc cupped (90 eyes)	0% (*n* = 18)	31% (*n* = 32)	6% (*n* = 34)	0% (*n* = 6)	13% (*n* = 90)
Retinopathy (86 eyes)	43% (*n* = 14)	50% (*n* = 32)	6% (*n* = 34)	50% (*n* = 6)	31% (*n* = 86)
Visual evoked potential delay (38 eyes)	80% (*n* = 10)	67% (*n* = 12)	50% (*n* = 4)	100% (*n* = 12)	79% (*n* = 38)

**Table 2:** The demographic data and ophthalmologic characteristics of nine Taiwanese patients with MPS I

No.	MPS type	Gender	Age (years)	Right eye or left eye	Visual acuity	Sphere	Cylinder	Axis of cylinder	Refraction (spherical equivalent)	Amblyopia	Corneal opacity	Lens opacity	Optic disc	IOP (mmHg)	Retinal appearance	VEP
I‐1	IH	F	8.3	R	0.6 (6/10)	+1.5	+0.5	150	+1.75	Y	+	Normal	Mild swelling	‐	Normal	Bilateral delay
				L	0.5 (6/12)	+2.0	+0.75	450	+2.38							
I‐2	IH/S	F	1.6	R	Follow light	‐	‐	‐	‐	N	++	Normal	Normal	‐	Normal	‐
				L												
I‐3	IH/S	F	2.2	R	Follow light	No target	No target	No target	No target	N	+	Normal	Normal	‐	Normal	Normal
				L		+2.25	−4.0	170	+0.25							
I‐4	IH/S	M	18.2	R	ND	‐	‐	‐	‐	N	++	‐	Normal	32.7	Poor view	Bilateral no response
				L	ND									28.3		
I‐5	IH/S	M	18.9	R	HM/20 cm	No target	No target	No target	No target	N	+++	Total opacity	Swelling	58	Poor view	R mild delay
				L	ND/10 cm	+7.25	+1.75	70	+8.13			Slight opacity	Normal	62		
I‐6	IS	M	13.2	R	1.0 (6/6)	+2.25	+2.25	85	+3.38	N	+	Nuclear sclerosis	Normal	18.1	RPE change	‐
				L	0.9 (6/6.7)	+2.00	+2.25	90	+3.13					14.3		
I‐7	IS	F	22.1	R	0.4 (6/15)	+5.0	+2.0	90	+6.00	Y	+	Normal	Normal	14	Foldings around macula	‐
				L	0.6 (6/10)	+5.25	+2.25	95	+6.38					13.5		
I‐8	IS	M	32.3	R	0.4 (6/15)	+2.0	+1.5	90	+2.75	N	+	Normal	Normal	15.8	RPE change	Bilateral mild delay
				L	0.7 (6/8.6)	+2.0	+2.0	90	+3.00					20		
I‐9	IS	M	34.9	R	0.8 (6/7.5)	+9.0	+1.5	140	+9.75	N	+	Nuclear sclerosis	Normal	14.3	Normal	‐
				L	0.3 (6/20)	+9.0	+1.5	90	+9.75	Y				15.3		

*Notes*. MPS, mucopolysaccharidosis; IOP, intraocular pressure; VEP, visual evoked potential; H, Hurler; H/S, Hurler/Scheie; S, Scheie; F, female; M, male; R, right; L, left; ND, digit number; HM, hand motion; ‐, not assessed; Y, yes; N, no; +, mild corneal clouding; ++, moderate corneal clouding; +++, severe corneal clouding; RPE, retinal pigment epithelial.

**Table 3:** The demographic data and ophthalmologic characteristics of 17 Taiwanese patients with MPS II

No.	MPS type	Gender	Age (years)	Right eye or left eye	Visual acuity	Sphere	Cylinder	Axis of cylinder	Refraction (spherical equivalent)	Amblyopia	Corneal opacity	Lens opacity	Optic disc	IOP (mmHg)	Retinal appearance	VEP
II‐1	II (S)	M	3.3	R	Follow light	‐	‐	‐	‐	N	Clear	Normal	Normal	‐	Normal	‐
				L												
II‐2	II (M)	M	6.0	R	0.8 (6/7.5)	+0.25	−0.25	29	+0.13	N	Clear	Normal	Cupped 0.4 cup:disc	18	Normal	‐
				L	1.0 (6/6)	−0.25	−0.25	4	−0.38					19		
II‐3	II (M)	M	6.6	R	0.9 (6/6.7)	+1.25	−3.0	180	−0.25	N	Clear	Nuclear sclerosis	Normal	23.3	Normal	‐
				L	0.9 (6/6.7)	+0.5	−2.5	170	−0.75					18.9		
II‐4	II (M)	M	6.7	R	0.7 (6/8.6)	‐	‐	‐	‐	N	Clear	Normal	Normal	21	RPE change	Normal
				L	0.7 (6/8.6)									21		
II‐5	II (M)	M	6.9	R	1.0 (6/6)	+1.0	−2.75	20	−0.38	N	Clear	Normal	Normal	‐	Normal	Normal
				L	0.7 (6/8.6)	+1.5	−2.0	160	+0.5							
II‐6	II (M)	M	7.2	R	1.0 (6/6)	+0.25	−0.25	155	+0.13	N	Clear	Normal	Normal	‐	Normal	‐
				L	1.0 (6/6)	−0.25	−0.5	20	−0.50							
II‐7	II (M)	M	10.7	R	‐	‐	‐	‐	‐	N	Clear	Normal	‐	‐	‐	Bilateral delay
				L												
II‐8	II (M)	M	15.4	R	0.4 (6/15)	+4.0	+1.0	100	+4.50	N	Clear	Normal	Cupped 0.4 cup:disc	28.5	Normal	‐
				L	0.7 (6/8.6)	+3.25	+1.25	80	+3.88					29.5		
II‐9	II (M)	M	16.0	R	0.9 (6/6.7)	+1.0	+1.5	100	+1.75	N	Clear	Nuclear sclerosis	Normal	‐	RPE change, macular puckering	‐
				L	0.7 (6/8.6)	+1.25	+2.0	80	+2.25							
II‐10	II (M)	M	16.5	R	0.6 (6/10)	+0.5	+2.0	105	+1.50	Y	Clear	Normal	Normal	21	RPE change	‐
				L	0.7 (6/8.6)	+2.75	−2.75	170	+1.38	N				16		
II‐11	II (M)	M	16.6	R	0.4 (6/15)	+1.5	−4.5	10	−0.75	Y	Clear	Normal	Cupped 0.7 cup:disc	26	RPE change; generalized nerve fiber layer depression	Bilateral delay
				L	0.4 (6/15)	+2.0	−4.5	166	−0.25	N			Cupped 0.6 cup:disc	26		
II‐12	II (M)	M	18.5	R	1.0 (6/6)	+1.5	−1.75	5	+0.63	N	Clear	Normal	Normal	25.3	RPE change	Bilateral delay
				L	0.9 (6/6.7)	0	+1.5	75	+0.75					21.8		
II‐13	II (M)	M	18.7	R	LP	+10.75	−2.5	170	+9.50	N	Clear	Normal	Swelling	‐	RPE change	‐
				L	NLP	+9.25	−2.25	5	+8.13							
II‐14	II (M)	M	19.8	R	0.7 (6/8.6)	+0.75	+1.75	89	+1.63	N	Clear	Normal	Cupped 0.5 cup:disc	22.7	RPE change	‐
				L	0.7 (6/8.6)	+1.0	+1.25	82	+1.63				Cupped 0.7 cup:disc	21.2		
II‐15	II (M)	M	21.7	R	‐	+4.0	−2.5	38	+2.75	N	Clear	Cortical opacity	Cupped 0.6 cup:disc	26.3	Normal	R mild delay
				L		+4.25	−2.5	165	+3.00					21.2		
II‐16	II (M)	M	27.9	R	1.0 (6/6)	+3.0	+1.0	99	+3.50	N	Clear	Normal	Normal	‐	Normal	‐
				L	1.2 (6/5)	+3.75	+1.5	87	+4.50							
II‐17	II (M)	M	34.3	R	0.6 (6/10)	+2.75	0	‐	+2.75	N	Clear	Normal	Swelling	12	RPE change	‐
				L	0.5 (6/12)	+3.25	+0.75	‐	+3.63					15		

*Notes*. MPS, mucopolysaccharidosis; IOP, intraocular pressure; VEP, visual evoked potential; (S), severe form; (M), mild form; M, male; R, right; L, left; LP, light perception; NLP, no light perception; ‐, not assessed; Y, yes; N, no; RPE, retinal pigment epithelial.

**Table 4:** The demographic data and ophthalmologic characteristics of 17 Taiwanese patients with MPS IV

No.	MPS type	Gender	Age (years)	Right eye or left eye	Visual acuity	Sphere	Cylinder	Axis of cylinder	Refraction (spherical equivalent)	Amblyopia	Corneal opacity	Lens opacity	Optic disc	IOP (mmHg)	Retinal appearance	VEP
IV‐1	IVA	M	1.1	R	Follow light	+0.5	−1.5	180	−0.25	N	+	Normal	Normal	‐	Normal	‐
				L	Follow light	+0.5	−1.5	180	−0.25							
IV‐2	IVA	M	5.6	R	0.2 (6/30)	+3.0	−3.0	13	+1.50	N	+	Normal	Normal	20.1	Normal	‐
				L	0.1 (6/60)	0	+3.0	80	+1.50	Y				17.5		
IV‐3	IVA	M	5.8	R	0.6 (6/10)	0	+3.0	89	+1.50	N	+	Normal	Normal	‐	Normal	‐
				L	0.6 (6/10)	+0.5	+2.25	91	+1.63							
IV‐4	IVA	F	6.4	R	0.2 (6/30)	+2.5	+2.5	85	+3.75	N	+	Nuclear sclerosis	Normal	21	Normal	‐
				L	0.3 (6/20)	+2.5	+2.5	90	+3.75					18		
IV‐5	IVA	F	6.5	R	0.4 (6/15)	−0.75	−1.5	1	−1.50	N	+	Normal	Normal	‐	Normal	‐
				L	0.4 (6/15)	0	−0.75	25	−0.38							
IV‐6	IVA	M	7.2	R	0.4 (6/15)	+0.75	+2.5	90	+2.00	Y	+	Normal	Normal	17	Normal	‐
				L	0.4 (6/15)	+0.75	+2.5	84	+2.00					15		
IV‐7	IVA	M	9.0	R	0.5 (6/12)	+0.25	+0.25	52	+0.38	N	+	Normal	Normal	‐	Normal	‐
				L	0.7 (6/8.6)	+0.25	+1.75	37	+1.13							
IV‐8	IVA	F	10.9	R	1.0 (6/6)	+0.25	−0.75	162	−0.13	N	+	Normal	Normal	‐	Normal	‐
				L	1.0 (6/6)	+0.5	−1.25	180	−0.13							
IV‐9	IVA	M	11.6	R	0.6 (6/10)	−1.0	−0.5	110	−1.25	N	Clear	Normal	Normal	‐	Normal	‐
				L	0.6 (6/10)	−1.0	−0.25	73	−1.13							
IV‐10	IVA	F	13.6	R	0.3 (6/20)	+3.5	−5.0	170	+1.00	N	+	Normal	Normal	‐	Normal	‐
				L	0.4 (6/15)	+4.5	−5.0	5	+2.00							
IV‐11	IVA	F	14.2	R	0.3 (6/20)	+2.5	−3.5	169	+0.75	Y	+	Normal	Normal	‐	Normal	‐
				L	0.4 (6/15)	+3.0	−4.5	177	+0.75							
IV‐12	IVA	F	15.7	R	0.3 (6/20)	+1.0	−1.75	140	+0.13	N	+	Normal	Normal	26.9	Normal	‐
				L	0.9 (6/6.7)	−0.25	−0.75	26	−0.63					27		
IV‐13	IVA	F	15.8	R	0.5 (6/12)	+3.0	−3.0	180	+1.50	N	++	Normal	Normal	‐	Vitreous opacities	‐
				L	0.4 (6/15)	+6.0	−5.0	175	+3.50							
IV‐14	IVA	M	15.9	R	1.0 (6/6)	+2.25	+1.00	65	+2.75	N	+	Normal	Normal	15.2	Normal	Normal
				L	0.9 (6/6.7)	+2.25	+1.5	98	+3.00					14.3		
IV‐15	IVA	F	19.2	R	0.8 (6/7.5)	−2.25	−2.25	150	−3.38	N	+	Normal	Cupped 0.5 cup:disc	25.1	Normal	‐
				L	0.4 (6/15)	−1.75	−1.75	55	−2.63					24.4		
IV‐16	IVA	M	25.5	R	0.5 (6/12)	−0.5	−1.5	170	−1.25	N	+	Normal	Normal	20.2	Normal	L borderline delay
				L	0.1 (6/60)	−2.25	−1.0	30	−2.75					18.3		
IV‐17	IVA	M	29.4	R	0.5 (6/12)	‐	‐	‐	‐	N	+	Normal	Normal	22	Normal	‐
				L	0.5 (6/12)									21		

*Notes*. MPS, mucopolysaccharidosis; IOP, intraocular pressure; VEP, visual evoked potential; F, female; M, male; R, right; L, left; ‐, not assessed; Y, yes; N, no; +, mild corneal clouding; ++, moderate corneal clouding.

**Table 5:** The demographic data and ophthalmologic characteristics of seven Taiwanese patients with MPS VI

No.	MPS type	Gender	Age (years)	Right eye or left eye	Visual acuity	Sphere	Cylinder	Axis of cylinder	Refraction (spherical equivalent)	Amblyopia	Corneal opacity	Lens opacity	Optic disc	IOP (mmHg)	Retinal appearance	VEP
VI‐1	VI	M	8.3	R	1.0 (6/6)	+5.0	+1.5	99	+5.75	N	+	clear	Normal	26.3	Normal	Bilateral mild delay
				L	0.8 (6/7.5)	+4.75	+2.0	81	+5.75					18.7		
VI‐2	VI	M	8.5	R	1.0 (6/6)	+2.25	+0.5	110	+2.50	N	+	normal	Mild swelling	18.4	Myelinated nerve fiber	Bilateral delay
				L	1.2 (6/5)	+1.5	+1.5	75	+2.25				Normal	14.8	Normal	
VI‐3	VI	M	9.0	R	0.01 (6/600)	+0.75	+1.0	95	+1.25	Y	+++	‐	‐	35	Poor view	Bilateral delay
				L	0.02 (6/300)	+2.25	−2.25	175	+1.13					37		
VI‐4	VI	F	11.7	R	0.2 (6/30)	+8.75	+0.5	101	+9.00	Y	++	clear	Swelling	17.7	Increased vessels tortuosity, vitrous opacities	Bilateral delay
				L	0.1 (6/60)	+8.5	+0.75	64	+8.88					19.5		
VI‐5	VI	F	13.0	R	HM/20 cm	‐	‐	‐	‐	Y	+++	‐	‐	38	Poor view	Bilateral delay
				L	HM/5 cm									39		
VI‐6	VI	F	21.4	R	HM/20 cm	‐	‐	‐	‐	Y	+++	‐	‐	25	Poor view	Bilateral delay
				L	HM/25 cm									26		
VI‐7	VI	F	25.6	R	0.1 (6/60)	‐	‐	‐	‐	Y	+++	‐	‐	26	Poor view	‐
				L	0.15 (6/40)									27		

*Notes*. MPS, mucopolysaccharidosis; IOP, intraocular pressure; VEP, visual evoked potential; F, female; M, male; R, right; L, left; HM, hand motion; ‐, not assessed; Y, yes; N, no; +, mild corneal clouding; ++, moderate corneal clouding; +++, severe corneal clouding.

### 3.1 Visual acuity and amblyopia

Among the 44 patients with available data for visual acuity, 15 patients (34%) had a visual acuity of less than 0.5 (6/12) equivalent in their better eye, including 71% of patients with MPS VI, 38% with MPS IV, 29% with MPS I, and 14% with MPS II. Among 100 eyes with a medical record of amblyopia evaluation, 22 eyes (22%) had amblyopia, including 71% of cases with MPS VI, 28% with MPS I, 15% with MPS IV, and 6% with MPS II.

### 3.2 Corneal opacity

**Figure 1:**
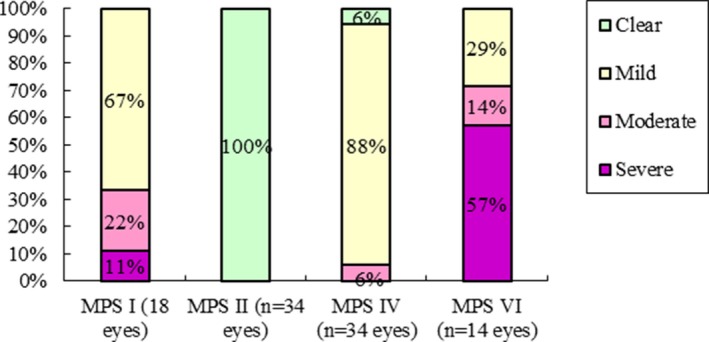
Severity of corneal opacity of different types of MPS (100 eyes). MPS, mucopolysaccharidosis

Among 100 eyes with a medical record of cornea condition, all patients with MPS I and VI and 94% of those with MPS IV had various degrees of corneal opacity, compared with all patients with MPS II having clear corneas. Severe corneal opacities were manifested in 57% of patients with MPS VI and 11% of MPS I patients, compared with none for MPS II and MPS IV patients (Figure 1).

### 3.3 Refractive error

**Figure 2:**
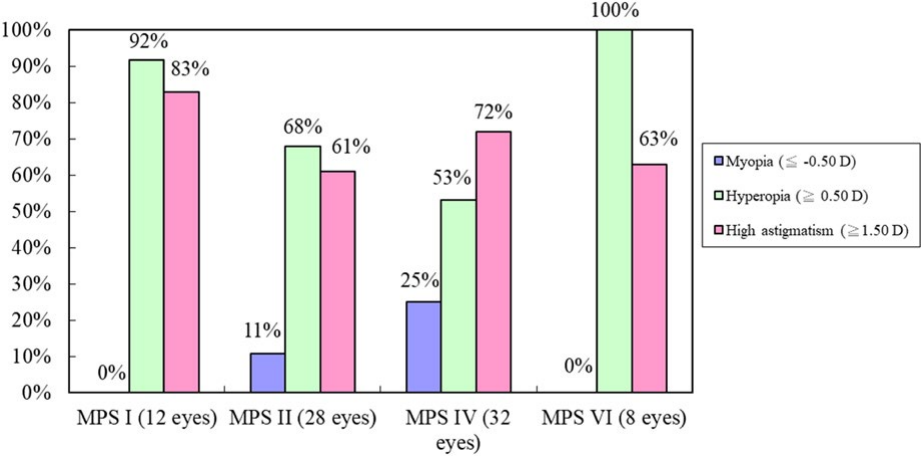
Refractive errors of different types of MPS (80 eyes), including myopia, hyperopia, and high astigmatism. Myopia is defined as ≦−0.50 D, hyperopia is defined as ≧0.50 D, and high astigmatism is defined as ≧1.50 D. MPS, mucopolysaccharidosis; D, diopters

Among 80 eyes with available data of refraction assessment, 11 eyes (14%) had myopia (≦−0.50 D), 55 eyes (69%) had hyperopia (≧0.50 D), as well as 55 eyes (69%) had high astigmatism (≧1.50 D). Twenty‐five percent of MPS IV patients had myopia, followed by 11% of MPS II cases. However, none of the patients with MPS I and MPS VI had myopia. All MPS VI cases had hyperopia, followed by 92% of MPS I patients, 68% of those with MPS II, and 53% of MPS IV patients. Eighty‐three percent of MPS I cases had high astigmatism, followed by 72% of patients with MPS IV, 63% with MPS VI, and 61% with MPS II (Figure 2).

### 3.4 IOP

**Figure 3:**
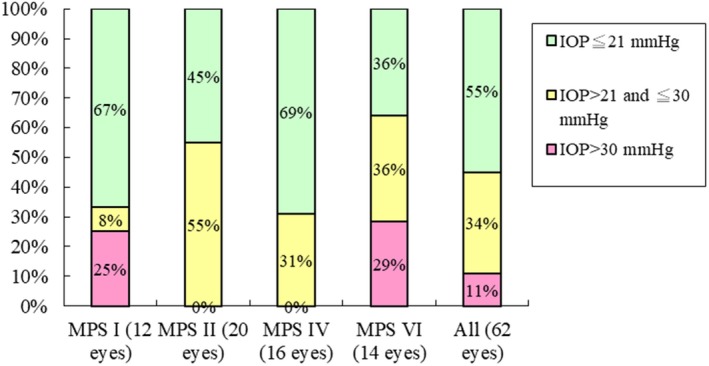
Intraocular pressure (IOP) of different types of MPS (62 eyes). IOP ≦ 21 mmHg is defined as the normal range, IOP > 21 mmHg is defined as ocular hypertension, and IOP > 30 mmHg is defined as severe ocular hypertension

**Figure 4:**
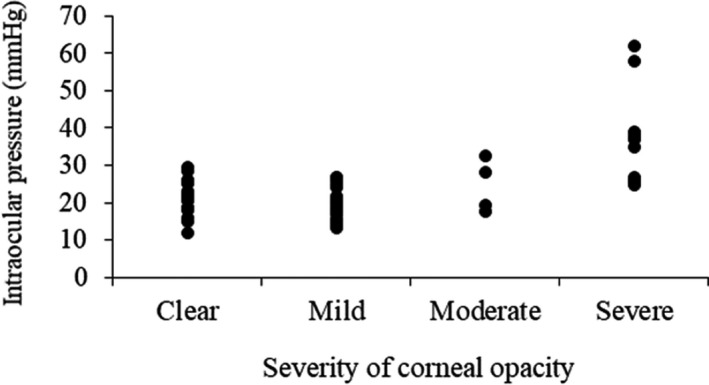
The relationship between intraocular pressure and the severity of corneal opacity for 62 eyes of mucopolysaccharidoses (*r *= 0.555, *p* < 0.01)

Among 62 eyes with available data for IOP, ocular hypertension (IOP > 21 mmHg) was found in 45% (28/62) of eyes, including 64% of MPS VI cases, 55% with MPS II, 33% with MPS I, and 31% with MPS IV. Severe ocular hypertension (IOP > 30 mmHg) was identified in 11% (7/62) of eyes, including 29% with MPS VI and 25% with MPS I, compared with no patients having MPS II and MPS IV (Figure 3). In this cohort, IOP was positively correlated with the severity of corneal opacity (*p *<* *0.01) (Figure 4).

### 3.5 Lens opacity

Among 90 eyes with available data for lens condition, lens opacity was found in 16% (14/90) of eyes, including 38% of the patients with MPS I, 18% with MPS II, 6% with MPS IV, and none with MPS VI.

### 3.6 Optic disc

Among 90 eyes with available data for optic disc condition, optic disc cupped was identified in 13% (12/90) of the eyes, including 31% with MPS II, 6% with MPS IV, and none of MPS I and MPS VI.

### 3.7 Retinopathy

**Figure 5:**
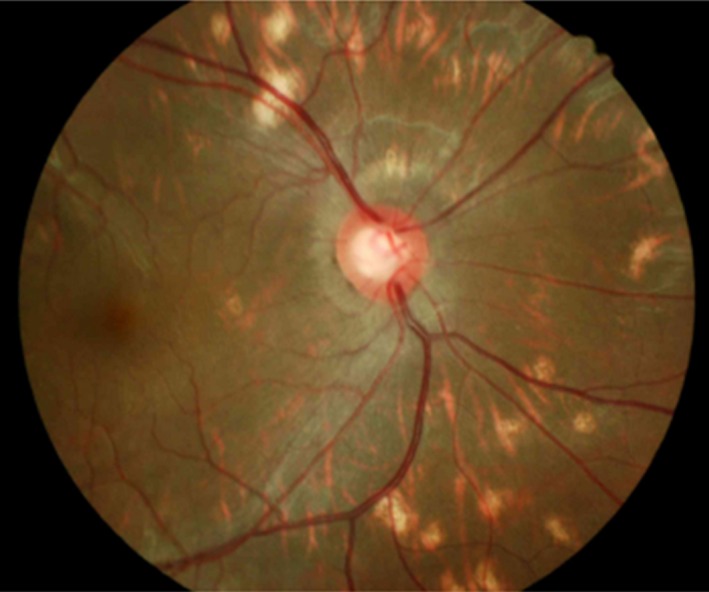
Retinal pigment epithelial change and optic disc cupped with cup‐to‐disc ratio of 0.7 (normal < 0.3) in a 16‐year‐old male patient with MPS II (patient No. II‐11)

Among 86 eyes with available data for retina condition, retinopathy (e.g., retinal pigment epithelial change) was found in 31% (27/86) of the eyes, including 50% with MPS II and VI, 43% with MPS I, and 6% with MPS IV (Figure 5).

### 3.8 VEP

Among 38 eyes with available data for VEP, 79% (30/38) showed VEP delay indicative of visual‐cortical pathway dysfunction, including all MPS VI patients, 80% with MPS I, 67% with MPS II, and 50% with MPS IV.

## 4 DISCUSSION

To the best of our knowledge, this is the first large cohort to describe the ophthalmologic features in Asian patients with MPS. There was limited original literature focusing on comprehensive ocular findings in different types of MPS (Ashworth, Flaherty, Pitz, & Ramlee, 2015; Ashworth et al., 2006b; Campos‐Campos, Pérez‐Torres, Villavicencio‐Torres, & González‐Vite, 2012; Collins, Traboulsi, & Maumenee, 1990; Couprie et al., 2010; Fahnehjelm et al., 2012; Suppiej et al., 2013; Villas‐Bôas, Fernandes Filho, & Acosta, 2011). Our study suggests that ocular complications with significant reduction in visual acuity are common in MPS patients. IOP was positively correlated with the severity of corneal opacity. Diagnostic problems may arise in these patients with severe corneal opacification, especially for those with MPS VI and MPS I. In this study, MPS VI and MPS I were identified to be the most severe types in a number of ophthalmologic manifestations among different types of MPS, including visual acuity, amblyopia, corneal opacity, hyperopia, ocular hypertension, optic disc cupped or swelling, retinopathy, and visual‐cortical pathway dysfunction. Our results are consistent with those of previous studies (Ashworth et al., 2006b, 2015; Campos‐Campos et al., 2012; Collins et al., 1990; Couprie et al., 2010; Fahnehjelm et al., 2012; Suppiej et al., 2013; Villas‐Bôas et al., 2011).

Visual impairment is common in patients with MPS due to their special ocular characteristics. Most patients experience gradual and irreversible vision loss, while some patients present rapid vision loss due to optic nerve swelling followed by atrophy or acute glaucoma. Other common conditions that affect vision in patients with MPS include amblyopia, hyperopia, and astigmatism. Visual impairment may also be caused by nonocular factors, including cortical visual pathway impairment (Ashworth et al., 2010; Summers & Ashworth, 2011). A case series including 50 patients with MPS by Ashworth et al. (2006b) using Snellen measurement described the visual acuity of less than 0.5 equivalent in their better eye was found in 79% of MPS I‐Hurler (MPS IH) cases, 44% with MPS I ‐Hurler‐Scheie (MPS IH/S), and 25% with MPS VI. In our study of 44 MPS patients, 34% had a visual acuity of less than 0.5 equivalent in their better eye, including 71% of MPS VI patients, 38% of those with MPS IV, 29% with MPS I, and 14% with MPS II.

Amblyopia (i.e. “lazy eye”) is a disorder of sight owing to the eye and brain not working well together leading to decreased visual acuity in an eye that otherwise typically appears normal. Ashworth et al. (2006b) described 32% (6/19) of patients with MPS IH, 33% (3/9) with MPS IH/S, 33% (1/3) with MPS I‐Scheie (MPS IS), none (0/2) with MPS II, and 25% (4/16) with MPS VI had amblyopia. In our study, overall 22% (22/100) of eyes had amblyopia, including 71% of those with MPS VI, 28% with MPS I, 15% with MPS IV, and 6% with MPS II. Our results were similar to theirs. Amblyopia has three main types, including strabismic, refractive, and deprivational types. Due to the limitation of the study design, the type of amblyopia was not available in this study.

Pastores et al. (2007) reported that corneal clouding was present in more than 80% in 302 patients enrolled in MPS I registry. Ashworth et al. (2006b) described that all their MPS I patients (*n* = 31) had some degree of corneal opacity. Both two MPS II patients had clear cornea. Thirty‐one percent of MPS VI patients (*n* = 16) had mild corneal opacity, 25% had moderate, and 38% severe. In our study of 100 eyes, all MPS I patients had certain degree of corneal opacity, all MPS II patients had clear cornea, 29% of MPS VI patients had mild corneal opacity, 14% had moderate, and 57% severe. Our results agree with theirs.

Hyperopia may be postulated to be reduced refractive power due to a more rigid and flattened cornea, as well as shortening of the axial length and sclera thickening as a result of GAGs storage in the sclera (Fahnehjelm, Törnquist, & Winiarski, 2012; Schumacher, Brzezinska, Schulze‐Frenking, & Pitz, 2008). Previous literature described that hyperopia occurs in >90% of patients with MPS I (all subtypes) and MPS VI (Ashworth et al., 2006b; Fahnehjelm, Törnquist, Malm, & Winiarski, 2006; Pitz, Ogun, Arash, Miebach, & Beck, 2009; Pitz et al., 2007). Villas‐Bôas et al. (2011) reported 54% (14/26) of patients with MPS presented astigmatism. Couprie et al. (2010) reported 60% (12/20) of patients with MPS IV had astigmatism. In our study for 80 eyes with refraction assessment, 55 eyes (69%) had hyperopia, 11 eyes (14%) had myopia, and 55 eyes (69%) had high astigmatism. There was lacking literature describing myopia in MPS in the Caucasian population. Since myopia is highly prevalent in East Asian countries and both prevalence and severity of myopia increased rapidly over the past two decades in younger generations in Taiwan (Guo, Lin, Lin, & Cheng, 2012), myopia in Taiwanese MPS patients may be due to ethnic characteristics. Kleinstein et al. (2003) reported that the prevalence of refractive errors in the general Asian population was 18.5% with myopia, 6.3% with hyperopia, and 33.6% with astigmatism. In the present study, our patients with MPS had more prevalence of hyperopia (69% vs. 6.3%) and astigmatism (69% vs. 33.6%) than the general population. Therefore, for patients with refractive errors, including hyperopia, myopia, and astigmatism, the prescription of correct glasses is recommended to avoid or minimize the risk of amblyopia and strabismus (Fahnehjelm et al., 2012).

Measurement of IOP is usually difficult for MPS patients because of their physical and intellectual disabilities. Corneal thickening may cause falsely high IOP results. Ashworth et al. (2006b) described that there was a low incidence of ocular hypertension in their MPS I patients, with only two MPS I patients (6%) having raised IOP. They found that ocular hypertension was more common in those with MPS VI, with 38% having IOP > 21 mmHg, and 15% >30 mmHg. In our cohort of 62 eyes, 33% of MPS I patients had an IOP >21 mmHg, 65% with MPS VI had an IOP >21 mmHg, and 29% of those with MPS VI >30 mmHg. Our results revealed that ocular hypertension was more common in Taiwanese MPS patients compared with previous reports in Caucasian MPS patients. In addition, we also found IOP was positively correlated with the severity of corneal opacity with a statistically significant relationship (*p *<* *0.01), which was consistent with the findings by Ashworth et al. (2006b). However, corneal thickening due to GAG deposition may also lead to falsely high IOP reading (Ashworth et al., 2006b). Thus further larger cohort studies are needed to evaluate the relationships between IOP and corneal opacity in patients with MPS. For MPS patients with corneal clouding, we have to balance the chance of successful treatment outcome against the potential risks on the management of glaucoma for these patients (Fahnehjelm et al., 2012).

Lens opacity has been described in patients with MPS IVA (Couprie et al., 2010; Olsen, Baggesen, & Sjolie, 1993). However, there was lacking literature reporting lens opacity in MPS I and II patients, except HSCT for MPS I may be associated with the development of cataract (Ashworth et al., 2006b; Fahnehjelm, Törnquist, Olsson, & Winiarski, 2007). In our study of 90 eyes without HSCT for lens assessment, lens opacity was found in 16% of eyes overall, including 38% of MPS I cases, 18% of MPS II, 6% of MPS IV, and none for MPS VI.

The existence of corneal opacity may lead to difficulties in the assessment of optic disc. Collins et al. (1990) reported that optic nerve head swelling preceded the development of optic atrophy in MPS. In their cohort of 108 patients, optic nerve head swelling was observed in 57% of the eyes of patients with MPS IH, 43% with MPS IH/S, 0% with MPS IS, 20% with MPS II, 0% with MPS IV, and 42% with MPS VI. Our data for 90 eyes showed optic disc swelling found in 17% of those with MPS I, 13% with MPS II, 50% with MPS VI, and none of MPS IV. Our results are consistent with theirs. Since the high risk of rapid optic nerve damage especially in MPS VI in our MPS population, the importance of rapid evaluation and treatment is suggested.

Clinical retinal pigment epithelial changes suggestive of retinopathy was reported to occur to variable degrees in MPS I, II, III, IV, and VI (Ashworth et al., 2006b; Caruso et al., 1986; Dangel & Tsou, 1985). Caruso et al. (1986) described that the ophthalmoscopic signs were less striking than the electrophysiologic findings using electroretinography, and they were usually restricted to mild changes of the retinal pigment epithelium. However, electroretinographic reports were not available in our cohort of 86 eyes. By dilated fundal examination of the retina, we found 50% of MPS II and VI cases, 43% of MPS I, and 6% of MPS IV cases had retinal pigment epithelial changes and retinopathy.

VEPs revealed the functional integrity of central vision at any level of the visual pathway including occipital cortex, optic pathway, retina, and eye (Ashworth et al., 2010). Ashworth et al. (2006b) reported 44% of MPS IH patients and 57% of those with MPS VI were found with abnormal VEP findings. Similarly, in our study with 38 eyes, all MPS VI cases, 80% of those with MPS I, 67% of MPS II, and 50% of MPS IV patients had abnormal VEP.

### 4.1 Limitations

As a retrospective study for this rare genetic disorder in Taiwan, there is a lack of complete ophthalmologic data for all enrolled subjects. Since study patients had to be cooperative and capable of following instructions for some ophthalmologic examinations to be performed, no patient with MPS III was enrolled in the study. The age range of patients in our study was quite broad (1.1–34.9 years), and the number of patients was small. Despite these limitations, our results were consistent with those of other case series in the literature that reported a high prevalence of ocular impairment among MPS patients. Thus, further exploration of the issue in larger cohorts with longer follow‐up periods is warranted.

## 5 CONCLUSION

Ocular complications, including corneal clouding, refractive errors (e.g., myopia, hyperopia and high astigmatism), ocular hypertension, lens opacity, optic disc cupped or swelling, retinal pigment epithelial change, visual‐cortical pathway dysfunction, amblyopia with significant reduction in visual acuity are common in Taiwanese MPS patients. These patients require regular ophthalmologic evaluations for the early detection and management of their ocular complications. MPS VI and MPS I were associated with the most severe types of ophthalmologic manifestations among different types of MPS. IOP was positively correlated with the severity of corneal opacity. Diagnostic problems may arise in patients with severe corneal opacification, especially for those with MPS VI and MPS I. These findings and the follow‐up data can be used to develop quality of care strategies for such patients.

## CONFLICT OF INTEREST

The authors declare that they have no competing interests.
